# Is Posttranslational Folding More Efficient Than Refolding from a Denatured State: A Computational Study

**DOI:** 10.1021/acs.jpcb.3c01694

**Published:** 2023-05-18

**Authors:** Quyen V. Vu, Daniel A. Nissley, Yang Jiang, Edward P. O’Brien, Mai Suan Li

**Affiliations:** † Institute of Physics, Polish Academy of Sciences, Al. Lotnikow 32/46, 02-668 Warsaw, Poland; ‡ Department of Statistics, University of Oxford, Oxford OX1 3LB, U.K.; § Department of Chemistry, Pennsylvania State University, University Park, Pennsylvania 16802, United States; ∥ Bioinformatics and Genomics Graduate Program, The Huck Institutes of the Life Sciences, Pennsylvania State University, University Park, Pennsylvania 16802, United States; ⊥ Institute for Computational and Data Sciences, Pennsylvania State University, University Park, Pennsylvania 16802, United States; # Institute for Computational Sciences and Technology, Quang Trung Software City, Tan Chanh Hiep Ward, District 12, Ho Chi Minh City 700000, Vietnam

## Abstract

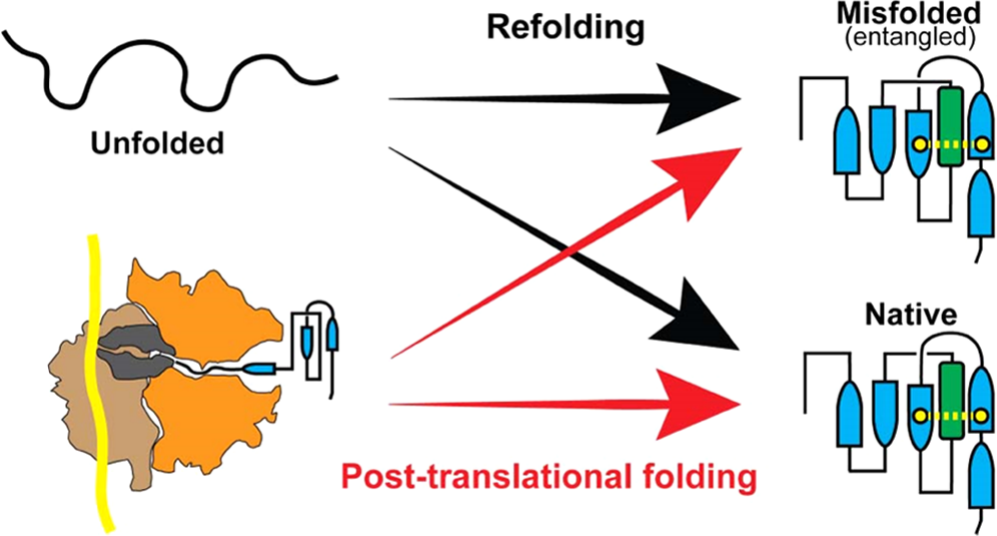

The folding of proteins into their native conformation is a complex process that has been extensively studied over the past half-century. The ribosome, the molecular machine responsible for protein synthesis, is known to interact with nascent proteins, adding further complexity to the protein folding landscape. Consequently, it is unclear whether the folding pathways of proteins are conserved on and off the ribosome. The main question remains: to what extent does the ribosome help proteins fold? To address this question, we used coarse-grained molecular dynamics simulations to compare the mechanisms by which the proteins dihydrofolate reductase, type III chloramphenicol acetyltransferase, and d-alanine–d-alanine ligase B fold during and after vectorial synthesis on the ribosome to folding from the full-length unfolded state in bulk solution. Our results reveal that the influence of the ribosome on protein folding mechanisms varies depending on the size and complexity of the protein. Specifically, for a small protein with a simple fold, the ribosome facilitates efficient folding by helping the nascent protein avoid misfolded conformations. However, for larger and more complex proteins, the ribosome does not promote folding and may contribute to the formation of intermediate misfolded states cotranslationally. These misfolded states persist posttranslationally and do not convert to the native state during the 6 μs runtime of our coarse-grain simulations. Overall, our study highlights the complex interplay between the ribosome and protein folding and provides insight into the mechanisms of protein folding on and off the ribosome.

## Introduction

Proteins are synthesized by ribosomes during the nonequilibrium process of translation and must fold to a specific native state, dictated by their amino acid sequence, to function. During translation, proteins are synthesized vectorially from N- to C-terminus based on an mRNA template. The nascent protein is initially confined to the ribosome exit tunnel, an ∼10 nm long tunnel with a diameter of 1–2 nm that can accommodate approximately 30 amino acids of the elongating protein.^1,2^ Due to its dimensions, the exit tunnel restricts the ability of the protein to self-interact and form a tertiary structure. However, many proteins fold cotranslationally^3−6^ as they begin to emerge from the exit tunnel and acquire a tertiary structure before their synthesis is complete. Though some small domains can fold inside the exit tunnel,^3−5^ most proteins can only begin to fold once they have left the exit tunnel.^7−10^ The nonequilibrium nature of protein synthesis means that the ability of a protein to fold cotranslationally can depend on the speed at which amino acids are added to the growing nascent chain.^11,12^ Refolding of a protein from its full-length denatured state, however, allows all segments of the protein to simultaneously fold without the restriction of the exit tunnel or the influence of translation kinetics. Bulk refolding thus presents the opportunity for the formation of a vast number of non-native contacts between amino acids. In general, cotranslational folding is thought to be a beneficial process that aids in the efficient folding of complex proteomes.^13−15^ The importance of cotranslational folding is highlighted by the recent experimental finding that one-third of *Escherichia coli* (*E. coli*) proteins are not able to refold in bulk solution after complete unfolding,^16^ suggesting that cotranslational folding is critical to their ability to reach their native state.

The folding of a small number of proteins has been experimentally and computationally studied on and off the ribosome.^17−22^ Evidence so far suggests that the role of the ribosome in folding is protein-specific. For example, structure-based models in combination with an arrest-peptide assay and cryo-EM experiments indicate that the folding of titin I27 is conserved on and off the ribosome.^21^ Similarly, experiments and molecular simulations of src SH3 show that its folding pathways are the same on and off the ribosome.^22^ On the other hand, Tanaka et al. used coarse-grained molecular simulation to study the role of the ribosome in guiding multidomain protein folding, finding that folding on the ribosome is more efficient compared to refolding.^18^ Dabrowski-Tumanski et al. computationally studied a deeply knotted protein and found that the ribosome plays a key role in knot formation.^20^ In terms of kinetics, single-molecule laser optical tweezer experiments have found that the arrested ribosome nascent chain complexes have reduced protein folding rates compared to folding in bulk.^17,23^ These studies mostly focus on small proteins (∼100 residues) folding on translationally arrested ribosomes. In vivo, many nascent proteins diffuse into the cytosol after synthesis; if folding is not completed on the ribosome, it may complete posttranslationally. Hence, the ribosome may only influence the formation of intermediate states, which nonetheless can change the outcome of folding.^24,25^ Given the relative paucity of experimental and computational data on the differences between folding on and off the ribosome for large proteins, we believe the influence of the ribosome on protein folding mechanisms remains an open question.

**Figure 1:**
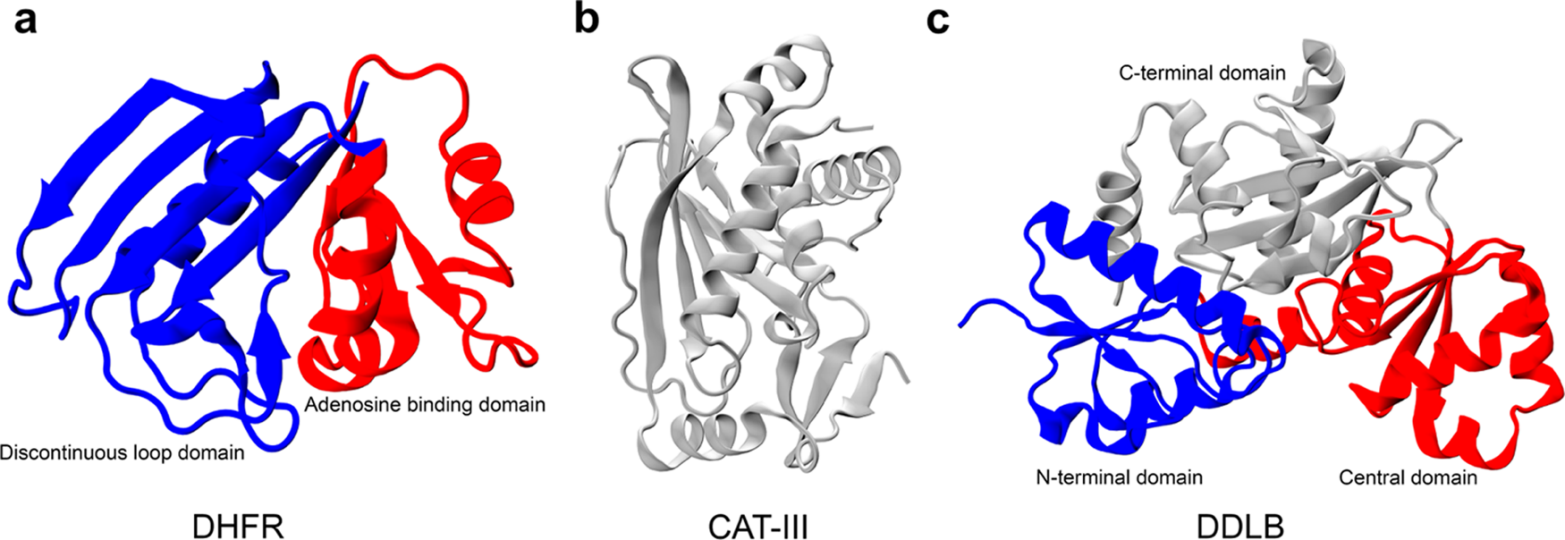
Crystal structures of three proteins in this study with domain-based coloring. (a) Crystal structure of DHFR (PDB ID: 4KJK); the discontinuous loop and the adenosine binding domains are shown in blue and red, respectively. (b) CAT-III is a single domain protein, which is shown in gray, and (c) DDLB protein (PDB ID: 4C5C), with the N-terminal, central, and C-terminal domains are shown in blue, red, and gray, respectively.

Performing all-atom folding simulations for large proteins is computationally infeasible. In this study, we, therefore, utilize a topology-based coarse-grained model to simulate the refolding in bulk solution as well as the co- and posttranslational folding of three *E. coli* enzymes (Figure 1): (i) dihydrofolate reductase (DHFR, 159 residues, PDB ID: 4KJK(26)), (ii) type III chloramphenicol acetyltransferase (CAT-III, 213 residues, PDB ID: 3CLA(27)), and (*iii*) d-alanine–d-alanine ligase B (DDLB, 306 residues, PDB ID: 4C5C(28)). DHFR, the smallest of the three, is composed of two domains.^29,30^ The adenosine binding domain (ABD) consists of residues 38–106, and the discontinuous loop domain (DLD) comprises residues 1–37 and 107–159 (Figures 1a and S1). DHFR catalyzes the NADPH-dependent reduction of dihydrofolate to tetrahydrofolate and has been a target enzyme of antifolate drugs.^31^ The native structure of CAT-III is composed of eight β-sheets and five α-helices (Figures 1b and S1); CAT-III is responsible for the high level of bacterial resistance to chloramphenicol.^32^ Finally, DDLB is a three-domain protein composed of an N-terminal domain (residues 1–85), central domain (residues 86–180), and C-terminal domain (residues 181–306), each of which is classified as α/β. At the secondary structure level, DDLB contains 10 β-sheets and 11 α-helices (Figures 1c and S1) and is an essential enzyme for the proper synthesis and maintenance of the bacterial cell wall.^33^

In this work, we apply multiple order parameters for protein folding, including the recently described entanglement parameter *G*, to investigate differences in folding on and off the ribosome. We find that while the ribosome assists the folding of DHFR, it does not promote the folding of CAT-III and DDLB, both of which contain a native entanglement. Our results support a mechanism by which the ribosome may promote the formation of intermediate misfolded states with non-native entanglements; these intermediates are kinetically trapped and persist for long time scales posttranslationally.

## Materials and Methods

### Simulation Details and Construction of Coarse-Grain Model

We employ a previously published Go̅-based coarse-grain model^11,34^ in which each amino acid is represented by a single interaction site placed at the C_α_ atom with a specific van der Waals radius for each amino acid; ribosomal RNA is represented as three or four beads per nucleotide, with one bead located at the phosphate position, another at the centroid of the ribose ring, and one at the centroid of each conjugated ring in the base (one bead for pyrimidine nucleobases and two beads for purine nucleobases). The potential energy of a configuration in this model is computed by the equation
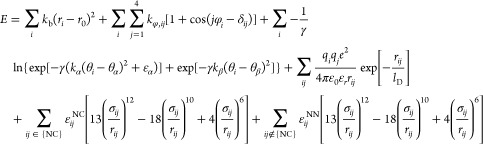
1The potential energy of a given conformation is calculated as a sum of the contributions from bonds, dihedral angles, bond angles, electrostatic interactions, Lennard-Jones-like native interactions, and repulsive non-native interactions. Model parameters are described in the previous studies.^11,34^ Parameters for three proteins in this study were taken from the previous work.^11^

In posttranslational folding simulations, we first performed continuous synthesis using the wild-type mRNA sequences, which are presented in Table S1. Synthesis simulations were conducted using a previously described protocol,^11,35^ with a cutout of the ribosome exit tunnel and surface. Codon-specific translation times were obtained from a previous study^11^ (Supplementary Table 8 of ref (11)). Once the protein sequence was fully synthesized, the covalent bond between the C-terminal site and the peptidyl transferase center (PTC) was cleaved and the protein was allowed to diffuse through the ribosome exit tunnel. Protein dissociation from the ribosome was defined as the point at which the position of the C-terminal residue was greater than 20 Å from the ribosome surface. At this point, the ribosome was removed and the left protein was able to undergo posttranslational folding in the absence of the ribosome.

The refolding simulations were initiated from the unfolded state, characterized by a low fraction of native contacts, *Q* value. Initial conformations for refolding simulations were generated by heating the native state of the protein to 1000 K for 15 ns. The final conformation from heating was then temperature-quenched at 310 K to initialize refolding. All simulations were carried out using a Langevin thermostat at a temperature of 310 K, with a time step of 15 fs and a friction coefficient of 0.050 ps^–1^. All simulations were carried out using OpenMM 7.7.^36^

In order to characterize protein folding, we conducted 200 statistically independent folding trajectories for each protein under investigation (100 trajectories of refolding and 100 trajectories of posttranslational folding). Each trajectory lasted for 6 μs, which corresponds to a real-time duration of approximately 24 seconds based on the relative acceleration of folding in these coarse-grain models relative to real time scales.^11,34^ For CAT-III and DDLB, which had a high prevalence of misfolded trajectories, we extended the simulation time to 30 and 15 μs, respectively, in order to determine if the proteins would eventually fold correctly in a longer time scale.

### Calculation of the Fraction of Native Contacts, ***Q***, and Its Usage to Determine Folded Trajectories

Two residues are considered to form a native contact if their α carbons are less than 8 Å apart in the crystal structure. To account for thermal fluctuations in contact distances during simulation, a flexibility parameter Δ = 1.2 was used: a native contact between two residues is classified to be formed in a current frame of the simulated trajectory if their distance is shorter than 1.2 times the distance in the crystal structure. The fraction of native contacts, *Q*, was calculated for each protein during their posttranslational folding or refolding simulations. Only contacts between pair of residues *i* and *j* both within secondary structural elements as identified by STRIDE^37^ and satisfying the criterion |*i* – *j*| > 3, where *i* and *j* are the residue indices, were considered; we excluded any secondary segment that is shorter than four residues from the analysis. To determine when a given trajectory of a protein is folded, we first characterized the fraction of native contact, *Q*, of each protein’s native state by performing ten 1.5 μs coarse-grained simulations at 310 K initialized from the native-state coordinates. The threshold for protein folding during refolding or posttranslational folding simulations, *Q*_threshold_, was determined as *Q*_threshold_ = ⟨*Q*_mode_^NS^⟩ – 3σ, where ⟨*Q*_mode_^NS^⟩ is the average *Q*_mode_ over all 15 ns windows of the ten 1.5 μs native-state simulations and σ is the standard deviation of ⟨*Q*_mode_^NS^⟩. To determine when folding occurred during refolding or posttranslational folding simulations, the mode of the *Q* values over a sliding 15 ns window was compared to the *Q*_threshold_. A given trajectory is defined as folded if during its time evolution, *Q*_mode_^15-ns^ ≥ *Q*_threshold_, the folding time is the first time that the above condition is met.^35,38^ The threshold value of *Q* for each protein is presented in Table 1.

**Table 1:** Threshold Value of *Q* of Three Proteins Computed from 10 Native-State Simulations Used to Determine if a Given Trajectory of Protein Folds

protein	*Q*_threshold_ = ⟨*Q*_mode_^NS^⟩ – 3σ
DHFR	0.9221
CAT-III	0.9269
DDLB	0.9521

### Estimating the Folding Time of Slow-Folding Proteins with a Large Proportion of Unfolded Trajectories

When the portions of folded trajectories are less than 50% of total trajectories, it is not possible to estimate the folding time as the median first passage time.

We consider three-state folding kinetics with parallel pathways. State A folds rapidly to the native state N at the rate *k*_1_, and state B folds slowly to the native state with a much smaller rate *k*_2_ (*k*_1_ ≫ *k*_2_), and there is no interconversion between A and B. We have a set of ordinary differential equations respecting the rate of changing portion of states A and B
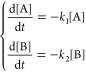
2where [A] and [B] are the portion of non-native states A and B. The portion (survival probability) of non-native states at time *t*: *S*_U_(*t*) = [A](*t*) + [B](*t*) = *c*_1_ exp(−*k*_1_*t*) + *c*_2_ exp(−*k*_2_*t*), where *c*_1_ and *c*_2_ are arbitrary constants. The initial condition that at time *t* = 0, the survival probability of non-native state = 1, we have *S*_U_ (*t* = 0) = *c*_1_ + *c*_2_ = 1, this yields: *c*_2_ = 1 – *c*_1_.

Hence, we computed the survival probability of the unfolded state as a function of time from simulations, and the resulting time series were then fit to the double-exponential equation
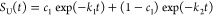
3*c*_1_, *k*_1_, and *k*_2_ are the fitting parameters. The time constants of the two kinetic phases are , with the larger of these two times determining the overall time scale of the folding process, τ_2_ ≫ τ_1_. To estimate the uncertainty of the folding time when fitting to double-exponential folding kinetics, we apply bootstrap resampling by randomly selecting trajectories from the list of simulations. We only consider the random sample with the coefficient of determination *R*^2^ > 0.9. This procedure was applied to estimate the folding time of CAT-III.

### Definition of the Progress Variable ς and Use to Monitor the Sequence of Pairs of Native Secondary Structure Elements Formed during the Folding Process

To account for the significant variation in folding times among different trajectories, we monitored folding pathways as a function of a progressive variable,^39^ ς, defined as
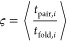
4where <···> indicates the average over all folded trajectories, and *t*_pair,*i*_ and *t*_fold,*i*_ are the folding time of pair and the whole protein folding time of the folded trajectory *i*, respectively. With this definition, we have 0 ≤ ς ≤ 1, ς = 0, which means that the pair under studied folds at the start of the simulation, and ς = 1 indicates the pair folds as the last step in the folding process. To determine the sequence of pairs of the secondary structure formation (defined in Figure S1 and Table S2), we consider a pair between two secondary structure elements that have more than one native contact. A pair is considered to be folded if its fraction of native contacts is larger than the threshold determined from native simulations. In our analysis of folding pathways, trajectories that did not fold within the 6 μs simulation duration were excluded.

### Identifying Entanglement and the Changes in Entanglement

We use the approximation to the partial Gaussian double integration method proposed by Baiesi and co-workers^40^ to calculate these partial linking numbers for a closed (loop) and open curve (termini). To identify lasso-like entanglements, we used the numerically invariant linking numbers,^41^ which describe the linking between a closed loop and an open segment in a three-dimensional space. This procedure is a modified version of the original protocol proposed by Baiesi to detect entanglement in coarse-grain protein structures. The original protocol is not computationally efficient to analyze trajectories since for each pair of contact, we have to calculate the linking number for all possible combinations of loop and threading segments. In our modified protocol, we only have to calculate the linking number between the closed loop (closes by native contact) and two tails. The closed loop is composed of the peptide backbone connecting residues *i* and *j* that form a native contact. Outside this loop is an N-terminal segment composed of residues 5 through *i* – 4 and a C-terminal segment composed of residues *j* + 4 through *N* – 5, where we exclude the first five residues of the N-terminal curve, the last five residues of the C-terminal curve, and four residues before and after the native contact to eliminate the error introduced by both the high flexibility and contiguity of the termini and trivial entanglements in the local structure; this metric is similar to whGLN.^42^ We can characterize the entanglement of each tail with the loop formed by the native contacts with two partial linking numbers denoted *g*_N_ and *g*_C_. For a given structure of an *N*-residue protein, with a native contact present at residues (*i*, *j*), the coordinates ***R***_*l*_ and the gradient d***R**_l_* of the point *l* on the curves were calculated as
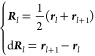
5where ***r***_*l*_ is the coordinates of the C_α_ atom in residue *l*. The linking numbers *g*_N_(*i*, *j*) and *g*_C_(*i*, *j*) were calculated as
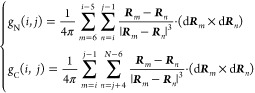
6The total linking number for a native contact (*i*, *j*) is therefore estimated as
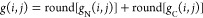
7Comparing the absolute value of the total linking number for a native contact (*i*, *j*) to that of a reference state allows us to detect a gain or loss of linking between the backbone trace loop and the terminal open curves as well as any switches in chirality. Therefore, there are six changes in linking cases we should consider when using this approach to quantify entanglement (see Supplementary Figure S1 and Table 1 of ref (43)).

The degree of entanglement *G* is defined as
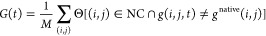
8where (*i*, *j*) is the native contact in the crystal structure; NC is the set of native contacts formed in the current structure at time *t*; and *g*(*i*,*j*,*t*) and *g*^native^ (*i*,*j*) are, respectively, the total linking number of the contact (*i*, *j*) at time *t* and native structures estimated using eq 7. M is the total number of native contacts in the native structure and Θ is a Heaviside step function, equals 1 if the condition is true and equals 0 if the condition is false.

The difference between *g*(*i*,*j*,*t*) and *G*(*t*) is *g*(*i*,*j*,*t*), which is characterized by the entanglement in a given structure of contact (*i*, *j*) at time t, while *G*(*t*) provided information about the total number of contacts that changed the entanglement at time *t*.

### Clustering and Coarse-Graining Conformational Space (***Q***, ***G***)

The projection of conformation space onto (*Q*,*G*) reveals intermediate states that may be hidden when projected onto *Q* alone, as two states can have the same value of *Q* but one may be entangled while the other is not. Entanglement can prevent a protein from reaching its native state, as the loop-threading segment is improperly organized. Entangled states thus can form kinetic traps with large energy barriers preventing progression to the folded state, as large sections of the protein must unfold to allow disentanglement. To derive the log probability surface as a function of (*Q*,*G*), we first combined (*Q*, *G*) data from refolding and posttranslational folding for each protein and applied the Min–Max algorithm^44^ for normalization. K-mean++ clustering^45^ was then utilized to identify microstates, with 200, 400, and 400 clusters (microstates) being used for DHFR, CAT-III, and DDLB, respectively. As k-mean++ is a distance-based clustering algorithm, the normalization of data was necessary to prevent one-order parameter from dominating the distance measure. The resulting clusters were further coarsened into a small number of metastable states using the PCCA+ algorithms^46^ to facilitate the interpretation of the folding pathways. The number of metastable states was determined based on the presence of a gap in the eigenvalue spectrum of the transition probability matrix; 11, 14, and 13 metastable states were used for DHFR, CAT-III, and DDLB, respectively. Both the clustering and coarse-graining processes were performed by using the PyEmma^47^ and Deeptime^48^ packages.

### Identify Folding Pathways along the Order Parameters (***Q***, ***G***)

To identify folding pathways from the simulated trajectories, the following procedure was followed:

(1) For each discrete trajectory, the starting state of the first frame is added to the pathway.
(2) The trajectory is then advanced, and the next state that differed from the last recorded state in the pathway was identified. If this state had not yet been recorded in the pathway, it was added to the pathway. If the state is already been recorded in the pathway, the pathway was truncated at the first instance of the recorded state and the trajectory was advanced from that point.
(3) Repeat Step (2) until the end of the trajectory is reached.

This process resulted in pathways that contained no loops, and only recorded the on-pathway states for each discrete trajectory. The distribution of distinct pathways and the probabilities of transitioning from one state to another was then estimated based on the pathways of all of the discrete trajectories. The initial, folded, and misfolded states (in the folding/misfolding pathways plots) are colored yellow, blue, and red, respectively. A state is considered misfolded if there is a trajectory that becomes trapped in that state, and there is no direct transition to the native state. The size of the nodes is proportional to the probability of the state appearing in the coarse-grained trajectories. The size of the edges connecting the nodes is proportional to the number of transitions between states, and the red number beside the edge is the total number of transitions observed in the coarse-grained trajectories.

### Back-Mapping the Coarse-Grained Model to an All-Atom Model for Visualization

To backmap the coarse-grained model to all-atom representation, the first step was to add coarse-grained interaction sites that represent the side-chain center of mass near the corresponding C_α_ beads. Then, the orientation of the side-chain center of mass beads was optimized through energy minimization while restraining the C_α_ positions. Next, Pulchra software^49^ was used to rebuild the nonhydrogen atoms of both the backbone and the side chain. Finally, additional energy minimization was performed in vacuum with position restraints applied to all C_α_ atoms to obtain the final all-atom structure.

## Results and Discussion

### DHFR Folds More Efficiently due to Protein Synthesis

To understand the influence of protein synthesis and the ribosome on the folding of DHFR, we constructed a topology-based coarse-grain model (see the Materials and Methods section) and simulated its folding through two different processes. First, we simulated protein refolding starting from a thermally unfolded ensemble. Second, to probe its folding when synthesized by the ribosome, we simulated continuous synthesis and posttranslational folding. This model has been previously shown to reproduce the cotranslational folding of HemK N-terminal domain,^2^ accurately predict changes in enzyme-specific activities,^11^ and to predict misfolded conformations of GlpD that qualitatively agree with LiP-MS experiments.^35^ To characterize the similarities and differences in how proteins reach the native state, we only analyzed the trajectories that resulted in successful folding.

**Figure 2:**
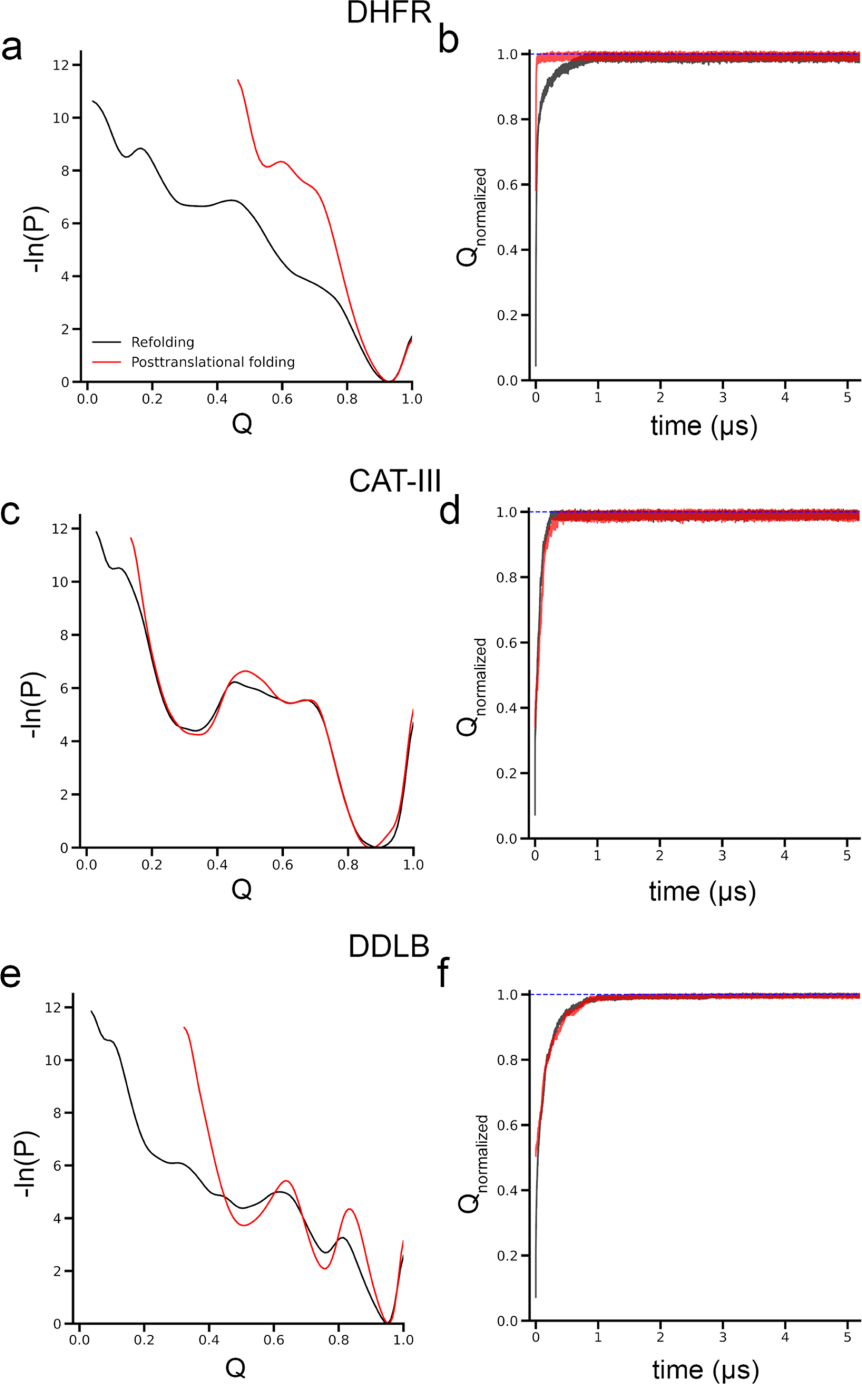
Influence of ribosomes on protein folding is protein-specific. Ribosome assists DHFR folding but does not promote CAT-III and DDLB folding. (a) Log probability landscape (−ln(*P*), where *P* is the probability of sampling a particular *Q* value) of DHFR, (b) average of the normalized fraction of native contacts, *Q*, of the folded trajectories as the function of time of DHFR; (c, d) same as in panels (a, b) but for CAT-III protein; and (e,f) same as in panels (a, c) but for DDLB. Refolding and posttranslational folding results are plotted in black and red colors, respectively. The blue-dashed lines in panels (b, d, f) indicate *Q*_normalized_ = 1.

We find that DHFR folds more efficiently when synthesized by the ribosome and undergoes posttranslational folding. However, when refolding from unfolded ensembles, some trajectories are trapped in misfolded states (*Q* < *Q*_threshold_) during the 6 μs of simulation time. Specifically, DHFR rapidly transitions from the initial structural ensemble to the folded ensemble. Since these simulations are out-of-equilibrium, we cannot speak of free-energy landscapes, which are time-independent; instead, we compute log probability landscapes (Figure 2a), which are time-dependent. This nonequilibrium landscape perspective for refolding and posttranslational folding simulations reveals differences between the two processes. DHFR has a well-defined structure composed of two main subdomains: the adenosine binding subdomain (ABD, residues 38–106) and the discontinuous loop subdomain (DLD, residues 1–37 and 107–159) (Figure 1a). In posttranslational folding simulations, this protein samples a smaller region of *Q* and the ABD domain folds cotranslationally and has the native form (*Q*_ABD_ = 0.98; Figure S2) at the start of posttranslational simulations. The DLD domain, consisting of both the N-terminus outside of the ribosome exit tunnel and the C-terminus, which is still within the exit tunnel, has a lower degree of native contacts *Q*_DLD_ = 0.27 (Figure S2). As a result, at the start of the posttranslational simulation, the overall structure of DHFR has approximately 60% of its native contacts formed, and the protein simply rearranges the DLD domain into the correct registry when the C-terminus is released from the exit tunnel. All trajectories reach the folded state (*Q* ≥ *Q*_threshold_ or *Q*_normalized_ ≥ 1) with a median folding time of 20.5 ns (95% confidence interval (CI) [18.5 ns, 24.8 ns], computed from bootstrapping). In contrast, refolding from the thermally unfolded ensemble involves initial conformations with a high degree of disorder (*Q* < 0.1 for both ABD and DLD domains; Figure S2), sampling a wider range of the log probability landscape (Figure 2a). Overall, the protein takes a longer time to reach the native state compared to posttranslational folding (Figure 2b), with a median folding time of 140.5 ns (95% CI [114.6 ns, 196.1 ns]) (Table 2). Only 92 (95% CI [86, 97]) trajectories fold out of 100 during the simulation. The difference between the median folding times is significant (*p*-value < 1 × 10^–6^, permutation test; Table 2), as well as the number of folded trajectories (*p*-value = 0.007; Table 2) between posttranslational folding and refolding. In both cases, the folding of DHFR proceeds with the ABD folding into its native form first, followed by the folding of the DLD (Figure S2). The folding of DLD is thus rate-limiting to the formation of the overall native structure.

**Table 2:** Folding Times and the Number of Folded Trajectories of Proteins in Refolding and Posttranslational Folding Simulations (95% Confidence Interval and *p*-Value Are Calculated from the Bootstrap Resampling and Permutation Test with 10^6^ Iterations)

	refolding		posttranslational folding	
protein	# folded trajectories [95% CI]	folding time (ns) [95% CI]	# folded trajectories [95% CI]	folding time (ns) [95% CI]
DHFR	92 [86, 97]	140.5 [114.6, 196.1]	100 [100, 100]	20.5 [18.5, 24.8]
	*p*-value (folded trajectories) = 0.007			
	*p*-value (folding time) < 10^–6^			
CAT-III	42 [32, 52]	2.3 × 10^5^ [6.5 × 10^4^, 1.7 × 10^12^]	31 [22, 40]	2.05 × 10^5^ [7.8 × 10^4^, 1.6 × 10^12^]
	*p*-value (folded trajectories) = 0.14			
	*p*-value (folding time) = 0.96			
DDLB	76 [67, 84]	522.5 [412.1, 712.2]	78 [70, 86]	426.3 [264.7, 690.9]
	*p*-value (folded trajectories) = 0.87			
	*p*-value (folding time) = 0.18			

### Protein Synthesis Does Not Increase the Folding Efficiency of CAT-III and DDLB

Using the same simulation protocol as DHFR, we performed refolding and posttranslational folding for CAT-III and DDLB proteins. In contrast to DHFR, the folding dynamics and population of folded trajectories for CAT-III and DDLB are relatively insensitive to posttranslational folding versus refolding. Specifically, for CAT-III, the log probability landscape of CAT-III is almost identical between posttranslational folding and refolding (Figure 2c). The progress of normalized *Q* of the folded trajectories is similar (Figure 2d), and the difference in the number of folded trajectories is insignificant (*p*-value = 0.14; Table 2). There are a large number of misfolded trajectories (*Q* < *Q*_threshold_; Table 2) within the simulation time of 6 μs. The proportion of folded trajectories for CAT-III is less than 50%; we, therefore, estimated its folding time by fitting the survival probability of the unfolded state as a function of time to a three-state kinetic model (eq 3; see Materials and Methods section). There is no statistical difference in folding times for CAT-III between refolding (2.3 × 10^5^ ns, 95% CI [6.5 × 10^4^ ns, 1.7 × 10^12^ ns]) and posttranslational folding (2.05 × 10^5^ ns, 95% CI [7.8 × 10^4^ ns, 1.6 × 10^12^ ns]), *p*-value = 0.96 (Table 2).

In the case of DDLB, more than 50% of trajectories are folded; hence, the median folding time could be estimated. We find that the median folding time in refolding is 522.5 ns (95% CI [412.1 ns, 712.2 ns]), compared to the folding time in posttranslational folding, which is 426.3 ns ([264.7 ns, 690.9 ns]). We find that there is no difference in the median folding times or the number of folded trajectories between the refolding and posttranslational folding simulations (*p*-value = 0.87 for the number of folded trajectories and *p*-value = 0.18 for the median folding time comparisons; Figure 2f and Table 2). However, there are some observed differences: the log probability landscape in the posttranslational folding of DDLB sampled a smaller region along the *Q* coordinate, and the local minima were deeper compared to refolding (Figure 2e). This suggests that the cotranslational formation of native contacts may have occurred after translation.

To test the influence of simulation time on the results, the misfolded trajectories for CAT-III were extended to 30 μs and the misfolded trajectories for DDLB were extended to 15 μs. We find that only one additional trajectory each from the refolding and posttranslational folding simulations of CAT-III folds during this extended duration, at 15 and 29.2 μs, respectively. No misfolded trajectories of DDLB folded in either the refolding or posttranslational folding simulations. This suggests that these misfolded trajectories are kinetically trapped and unlikely to convert to the folded state at longer time scales—consistent with previously published results.^11^

### Measuring the Folding Mechanisms of Proteins Using Progress Variable ς Reveals the Differences for DHFR and Remains Robust for CAT-III and DDLB

Protein folding is typically thought to occur in a hierarchical fashion, with secondary structural elements first forming individually and then cooperatively coalescing into tertiary structures. With this in mind, we characterize the folding process of DHFR, CAT-III, and DDLB as the temporal sequence of formation of their stable pairs of native secondary structural elements with the aid of a progress variable, ς (see the Materials and Methods section, eq 4). The value of ς is relative to the time of complete folding of the protein, with ς = 0 indicating that the pair folds at the start of the simulation and ς = 1 indicating the pair folds as the last step in the folding process. To simplify the analysis, we restrict ourselves to pairs of secondary structures that have more than one native contact, as described in the Materials and Methods section and Table S2.

**Figure 3:**
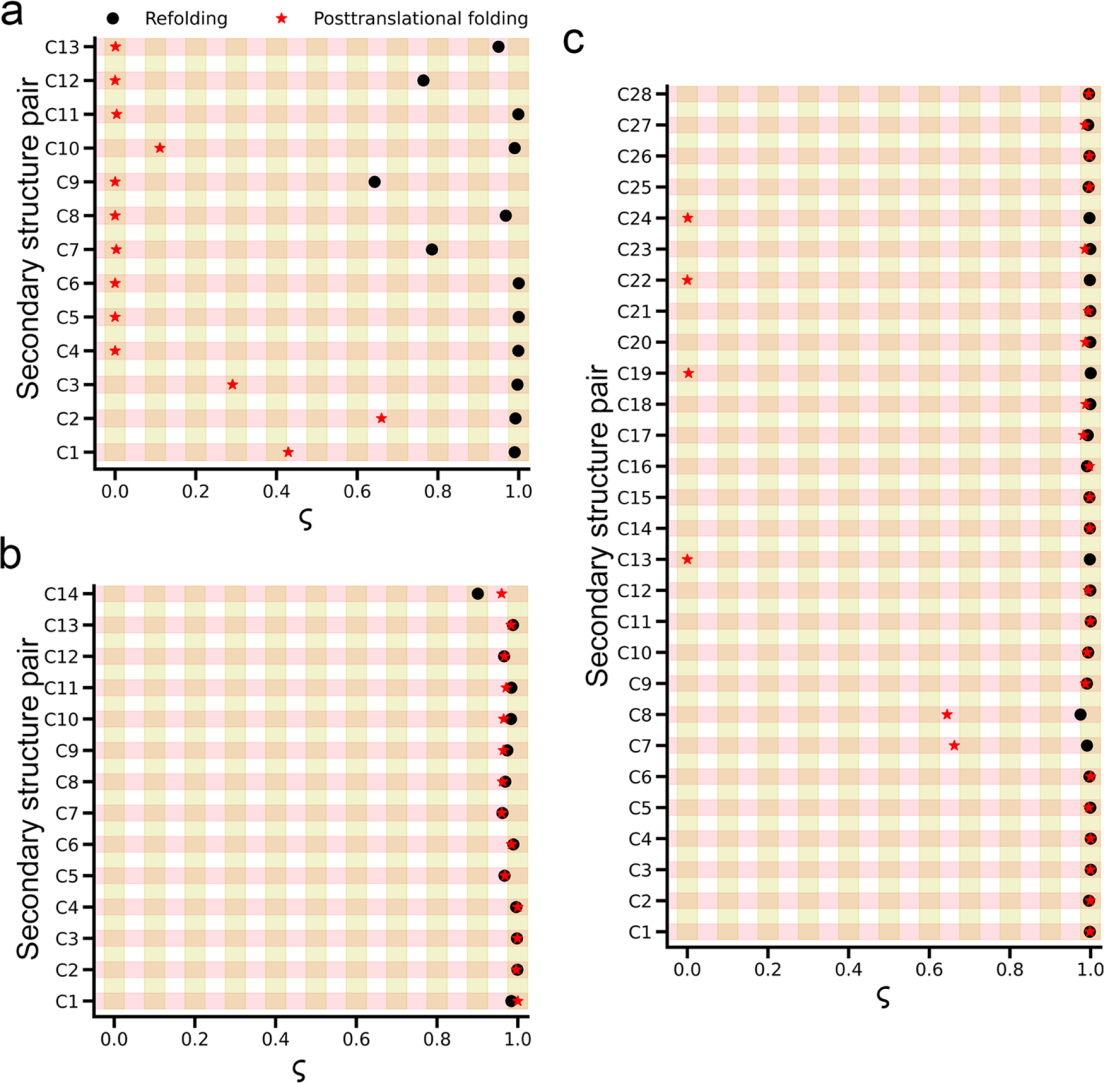
Comparisons of folding processes of DHFR, CAT-III, and DDLB in posttranslational folding versus refolding are shown as temporal sequences of secondary structure pairs formed over time with the aid of a progress variable ς. (a) Folding mechanism of DHFR is significantly different: all pairs in the ABD domain fold cotranslationally in posttranslational folding simulations, (b) CAT-III: there is no difference between posttranslational folding versus refolding, and (c) DDLB protein exhibits a small difference in four pairs of the center domain (C13, C19, C22, and C24) and two pairs (C7, C8) in the N-terminal domain. Refolding and posttranslational folding data are represented by black circles and red stars, respectively.

Based on this analysis, we observe a significant difference in DHFR. In posttranslational folding, all pairs of the native secondary structural elements belonging to the ABD domain fold cotranslationally (ς ∼ 0), while in refolding, most of the pairs fold at the end of the folding process (ς ∼ 1) (Figure 3a and Table 3). This suggests that the vectorial synthesis from the N-terminus to the C-terminus prevents the spontaneous cotranslational folding of some β-sheets in the C-terminal (C1, C2) and that the complete folding of DHFR occurs immediately upon release of the C-terminal from the ribosomal exit tunnel. These observations are consistent with previous experimental studies that have found that the central domain (ABD) acts as an independent folding unit during translation, while the DLD domain folds posttranslationally.^30^ For CAT-III, the sequence of secondary structure pair folding is similar in both refolding and posttranslational folding, with all pairs folding late during the folding process (ς ∼ 1; Figure 3b and Table 3). For DDLB, the overall folding order is similar, but some differences were observed, such as in posttranslational folding, four pairs in the center domain (C13, C19, C22, and C24) fold cotranslationally (ς = 0), two pairs in the N-terminal domain (C7, C8) fold posttranslationally but before the complete folding occurs (ς ∼ 0.65; Figure 3c and Table 3), while these pairs fold at the last event in refolding. Thus, protein synthesis and posttranslational folding do not significantly perturb the folding mechanisms of CAT-III and DDLB.

**Table 3:** Sequence of Native Secondary Structure Pair Formation during the Folding Process of DHFR, CAT-III, and DDLB Proteins^a^

protein	refolding	posttranslational folding
DHFR	C9 → C12 → C7 → (C8, C13) → (C1, C2, C3, C4, C5, C6, C10, C11)	(C4, C5, C6, C7, C8, C9, C11, C12, C13) → C10 → C3 → C1 → C2
CAT-III	C14 → (C5, C7, C8, C9, C12) → (C1, C2, C3, C4, C6, C10, C11, C13)	(C5, C7, C8, C9, C10, C11, C12, C14) → (C1, C2, C3, C4, C6, C13)
DDLB	(C1, C2, C3, C4, C5, C6, C7, C8, C9, C10, C11, C12, C13, C14, C15, C16, C17, C18, C19, C20, C21, C22, C23, C24, C25, C26, C27, C28)	(C13, C19, C22, C24) → (C7, C8) → (C1, C2, C3, C4, C5, C6, C9, C10, C11, C12, C14, C15, C16, C17, C18, C20, C21, C23, C25, C26, C27, C28)

a Pairs in parentheses represent secondary structures that are folded simultaneously.

### Native Entanglements Exist in the Crystal Structure of CAT-III and DDLB Proteins

We hypothesized that there is something distinct about the native topologies of CAT-III and DDLB that leads to a large proportion of misfolding. Several recent papers have predicted a link between misfolding involving a change in the entanglement status and long-lived misfolded states,^11,35^ including the failure to form native entanglements. Indeed, this is the molecular hypothesis explaining the observation that experimental folding rates of proteins decrease as the number of times the threading segment pierces the loop increases.^40^ To further understand this phenomenon, we investigate whether entanglement may play a role here by calculating the degree of entanglement for these proteins using eq 7.

**Figure 4:**
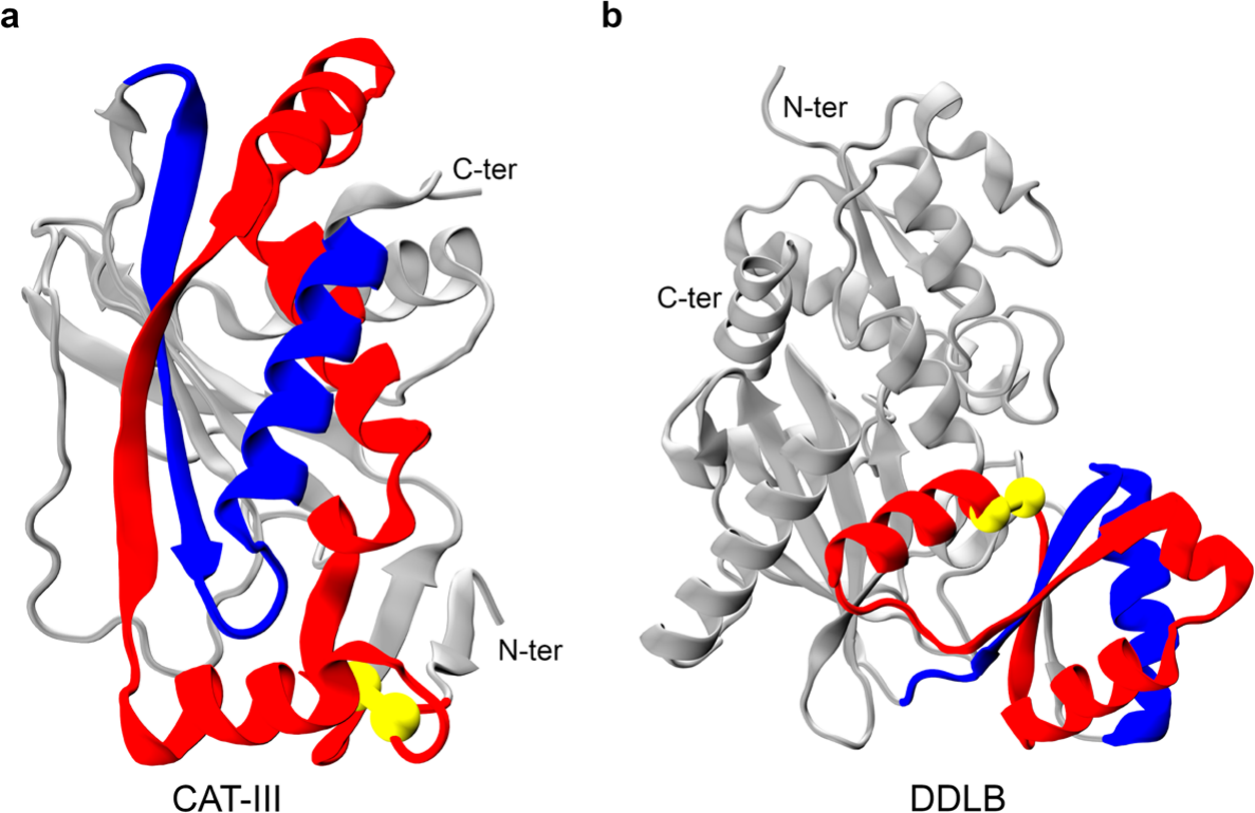
Example of native entanglements in the crystal structures of CAT-III and DDLB. The closed loop and crossing section of the threading segment of their entangled regions are colored red and blue, respectively. The loops are closed by noncovalent contacts between two residues (colored yellow), and the rest part of the protein is colored gray. (a) Representative native entanglement in CAT-III: the loop (colored red) is closed by a native contact between residues 8 and 77, and the threading segment consists of residues 177–208. (b) Representative native entanglement in DDLB: the loop (colored red) consists of residues 98–146, and the threading segment consists of residues 160–184.

We find that the crystal structure of DHFR does not contain any entanglements. In contrast, CAT-III has 16 native entanglements, with 14 of them consisting of a loop located near the N-terminus and a threading segment at the C-terminus. The remaining two native entanglements have a loop located near the C-terminus and a threading segment at the N-terminus. Similarly, DDLB has 36 native entanglements, half of which consist of a loop located closer to the N-terminus and a threading segment at the C-terminus, while the other half has a loop located closer to the C-terminus and a threading segment at the N-terminus. Representative examples of these entanglements are shown in Figure 4a,b for CAT-III and DDLB, respectively. Furthermore, proteins with an entanglement loop closer to the N-terminus were found to be folded more difficultly than the proteins with a loop closer to the C-terminus.^50^ This explains why DHFR (without native entanglement) can fold easily and small portions of CAT-III trajectories (most entanglement loops are located near the N-terminus) folds in our simulation. This observation suggests that entanglement plays an important role in the proper folding of proteins.

### Protein Synthesis Assists the Folding of DHFR by Avoiding Misfolded States with Non-Native Entanglements

**Figure 5:**
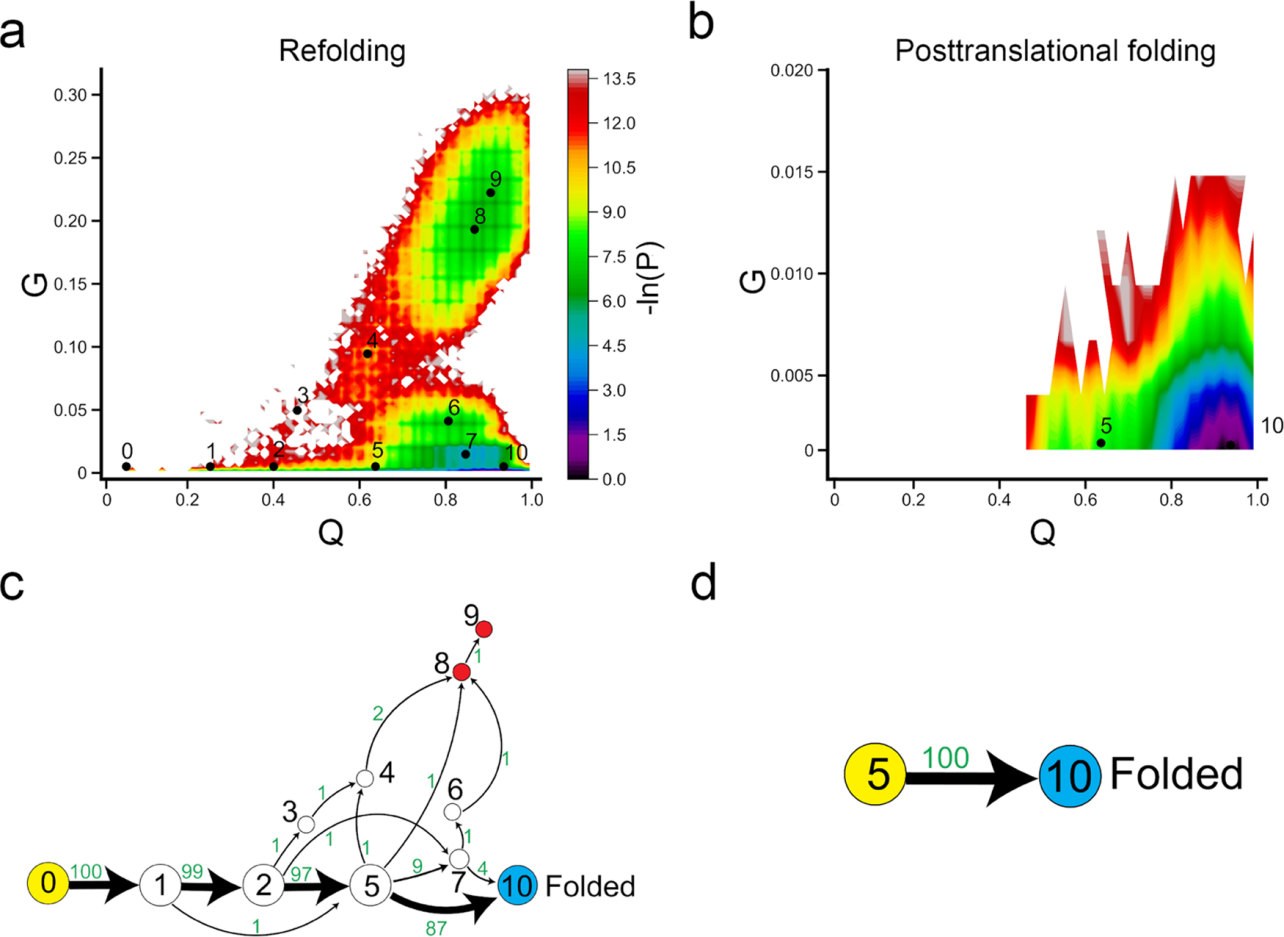
Ribosome helps DHFR fold more efficiently. (a, b) −ln(*P*) surface in refolding and posttranslational folding, respectively, where *P* is the probability of sampling particular *Q* and *G* values. The centers of metastable states and their corresponding indices are shown on top of the surface (black points). (c, d) Transition network from discrete trajectories of refolding and posttranslational folding simulations. The yellow, red, and sky blue nodes correspond to the initial, misfolded, and folded states, respectively. The black numbers on the nodes match the indices of metastable states in panels (a) and (b). The red numbers beside the edges indicate the number of direct transitions between states observed in discrete trajectories.

Entanglement plays an important role in the proper folding of proteins. To further characterize the folding pathways of DHFR, we clustered the conformational space based along the order parameters *Q* and *G* and then assigned them to metastable states (see the Materials and Methods section). In posttranslational folding, DHFR can spontaneously fold to its native state once the C-terminus is released from the ribosome. The two-dimensional log probability surface is concentrated in the region around the folded state (small *G*, high *Q*; Figure 5b), which is consistent with our 1D log probability landscape from the previous section. Specifically, the posttranslational folding simulations of DHFR only sample two states, 5 and 10 (the folded state). There are no misfolded trajectories in posttranslational folding, as all trajectories reach the folded state at the end of the simulation. The protein cotranslationally folds to the ensemble state 5, which has about 60% of native contacts formed (the fraction of native contacts with non-native entanglement is negligible, around 0.16%), and the folding process simply involves diffusion to the folded state (state 10). Folding network analyses reveal that 100% of folding pathways go straight from the initial state 5 to the folded state 10 (Figure 5d). There is no off-pathway state in the posttranslational folding of DHFR.

Refolding from the thermally unfolded ensemble is more complicated, compared to posttranslational folding. The −ln(*P*) surface has sampled a broad region in the non-native (low *Q*) or near-native (high *Q*) regions. We found that the population of DHFR refolding samples had a large number of entangled states, indicated by high values of *G* (Figures 5a and S3). The protein follows two parallel pathways to reach the native state: we find that the dominant pathway (*→ 5 → 10), which is the only pathway observed in posttranslational folding, accounts for 87% of the total trajectories in refolding simulation and a small portion (four trajectories, accounts for 4% of total trajectories) folds via intermediate state 7 (*→ 7 → 10). In addition, we find that 9% of trajectories become trapped in misfolded states (states 7–9). The broader −ln(*P*) surface in refolding is caused by a small number of misfolded trajectories. Five trajectories become trapped in state 7, three trajectories become trapped in state 8, and one trajectory becomes trapped in state 9. States 8 and 9 are off-pathway misfolded states, as we do not observe any folding events (conversion to the folded state 10) if the protein visits these states. When the protein samples the near-native state 7, only 40% of trajectories can fold successfully (* → 7 → 10/folded), while the remaining 60% fold to misfolded states (Figure 5c).

### Non-Native Entangled States Act as a Kinetic Trap in Both Refolding and Posttranslational Folding of CAT-III and DDLB

**Figure 6:**
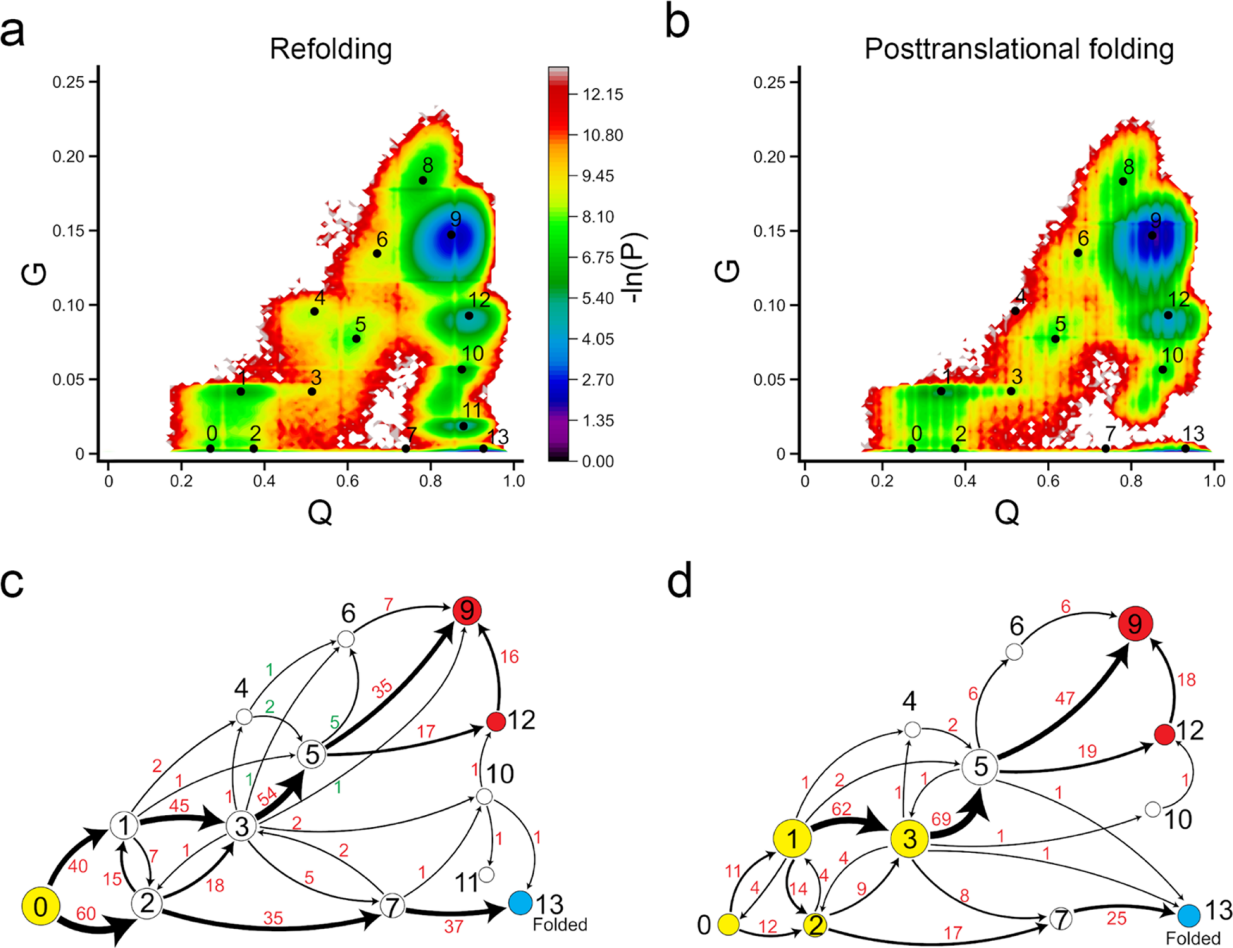
Protein synthesis does not increase the folding efficiency of CAT-III. (a, b) −ln(*P*) surface in refolding and posttranslational folding, respectively, where *P* is the probability of sampling particular *Q* and *G* values. The centers of metastable states and their corresponding indices are shown on top of the surface (black points). (c, d) Transition network from discrete trajectories of refolding and posttranslational folding simulations. The yellow, red, and sky blue nodes correspond to the initial, misfolded, and folded states, respectively. The black numbers on the nodes match the indices of metastable states in panels (a) and (b). The red numbers beside the edges indicate the number of direct transitions between states observed in discrete trajectories.

In contrast to DHFR, it seems that the ribosome has less effect on the folding/misfolding mechanism of CAT-III. The conformational space is very similar between refolding and posttranslational folding, and these two processes share almost all of the observed states (Figure 6). This is reasonable as we have observed that when the protein synthesis is completed, there is a small portion of native contacts that have been formed in CAT-III and hence can be considered an unfolded state (*Q* ∼ 30%; Figure 2d). Therefore, when the protein dissociates from the ribosome and undergoes posttranslational folding, this process is similar to folding from unfolded ensembles.

There are two critical classes of intermediate states in the folding of CAT-III: state 1, which leads to misfolding when some native contacts change entanglement, and state 2, which leads to the native state without changing entanglement. In posttranslational folding, a large number of trajectories initiate in state 1 (68%, then transition to state 3) and state 3 (9%), with some portions of native contacts changing entanglement. These trajectories mainly end up in misfolded states (states 9 and 12). Only 27% (CI 95% [19%, 36%]) of total trajectories can fold to the native state. In refolding, the process starts in the fully unfolded state 0 and diversifies to state 1 (40%), where some contacts change entanglement and lead to further misfolding, and a larger number of trajectories go to state 2 (60%) and then fold correctly to the native state. This results in slightly more folded trajectories in refolding (38%, CI 95% [30%, 49%]) compared to posttranslational folding. Thus, protein synthesis and posttranslational folding do not increase the folding efficiency of CAT-III compared to refolding but rather cause the protein to partially fold into misfolded intermediate states.

States 9 and 12 are likely long-lived misfolded states, as even when we extended the simulation time to 30 μs, we did not observe any misfolded trajectories folding to the native state (when considering both *Q* and *G* parameters). All of these misfolded states are near-native (high *Q*) and have a large number of native contacts changing entanglement (Figure S4).

**Figure 7:**
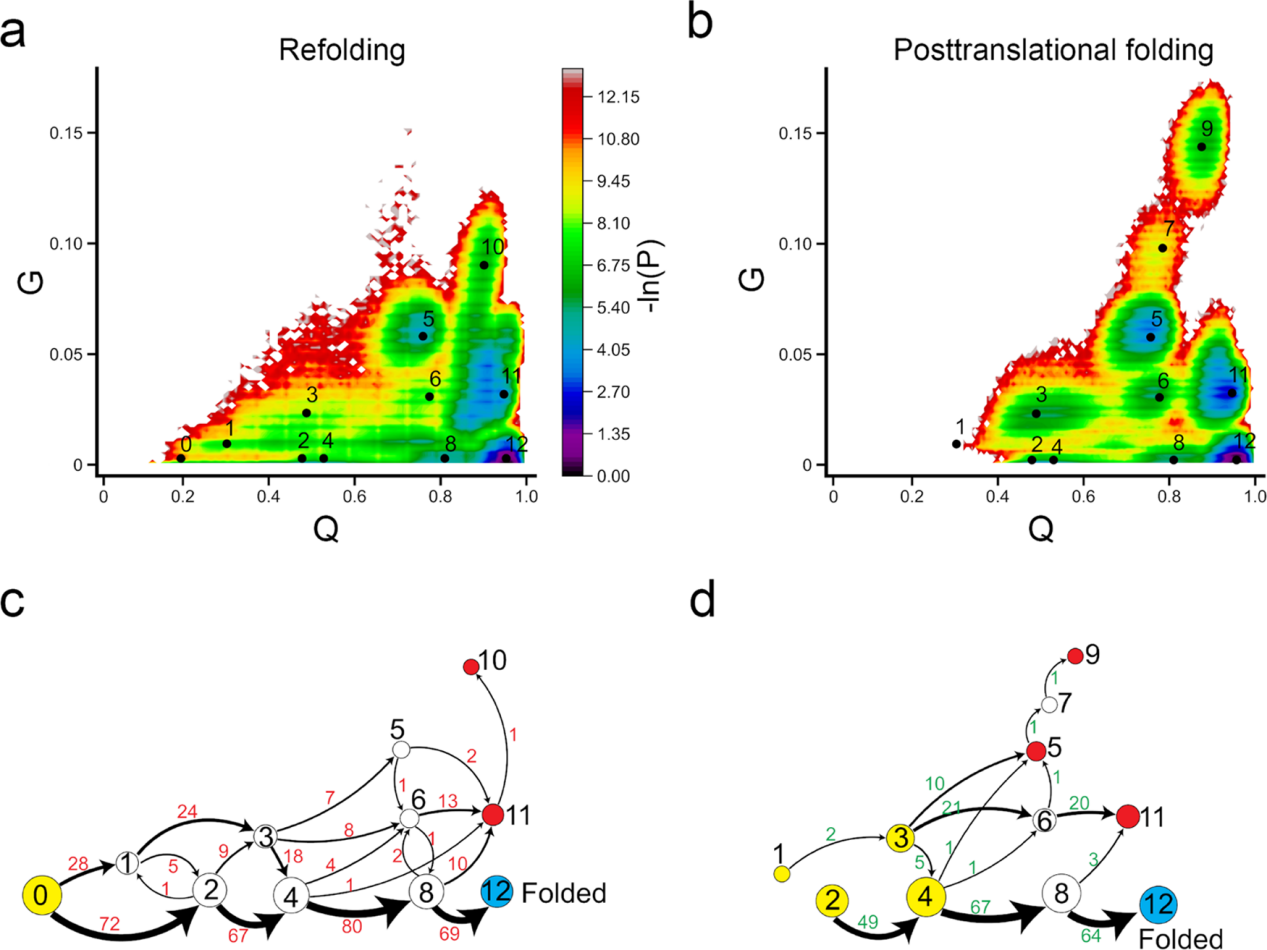
No difference in folding mechanisms of DDLB between refolding and posttranslational folding. (a, b) −ln(*P*) surface in refolding and posttranslational folding, respectively, where P is the probability of sampling particular *Q* and *G* values. The centers of metastable states and their corresponding indices are shown on top of the surface (black points). (c, d) Transition network from discrete trajectories of refolding and posttranslational folding simulations. The yellow, red, and sky blue nodes correspond to the initial, misfolded, and folded states, respectively. The black numbers on the nodes match the indices of metastable states in panels (a) and (b). The red numbers beside the edges indicate the number of direct transitions between states observed in discrete trajectories.

Similar to CAT-III, the ribosome does not aid in the proper folding of DDLB (Figure 7 and Table 4). Our simulations indicate that the overall −ln(*P*) surface is similar in refolding and posttranslational folding simulations. The dominant folding pathway is * → 2 → 4 → 8 → 12. In the posttranslational folding simulation, if the DDLB protein is in states 2 or 4 after protein synthesis (which occurs in 64% of trajectories), it has a high likelihood of successfully folding posttranslationally (2|4 → folded: 98.4%). On the other hand, if the protein is in states 1 or 3 after protein synthesis (36% of all trajectories in our simulations), it is likely to result in a misfolded state posttranslationally (1|3 → misfolded: 97.7%). This has also been observed experimentally for other proteins.^13,24,25^

**Table 4:** Percentage of Folding Pathways of DDLB in Refolding and Posttranslational Folding Simulations

pathways	percent (%)	pathways	percent (%)
Refolding			
0 → 1	28	1 → misfolded	67.9
0 → 2	72	2 → folded	83.3
Posttranslational folding			
cotranslational folding → 1|3	36	1|3 → misfolded	97.7
cotranslational folding → 2|4	64	2|4 → folded	98.4

Analysis of refolding pathways shows a similar distribution to posttranslational folding, with two classes of folding: one leading to correct folding (69%) and the other leading to misfolding (31%). In refolding simulations, proteins that start in a fully unfolded state (state 0) diversify into the intermediate misfolded state 1 (28% of transitions, with a change in entanglement) and remain trapped in misfolded states (1 → misfolded: 67.9%), while those that sink to state 2 mainly transition to the native state 12 (2 → folded: 83.3%).

State 10 is observed in refolding simulations but not in posttranslational folding, while states 7 and 9 are observed in posttranslational folding but not in refolding. These differences are exhibited in a single misfolded trajectory. In both refolding and posttranslational folding, we did not observe the transition from the near-native state 11 to the native state 12.

Overall, protein synthesis does not increase the folding efficiency of CAT-III and DDLB; intermediate states with non-native entanglement form cotranslationally and persist posttranslationally, and these states act as kinetic traps in protein folding. It should be noted that this work uses a “structure-based” model of protein folding, which encodes that the native state is the global minimum of free energy in our simulations; hence, misfolded states (i.e., those observed for CAT-III and DDLB) are metastable states and kinetically trapped, meaning that they have high free-energy barriers separated from the native state, making them convert to the native state very slowly. One possible limitation of our approach is that the non-native entangled states that we observed can be artifacts of our coarse-grained model. However, in a recent study, we showed that non-native entangled states also occur in all-atom simulations of proteins,^43^ suggesting that they are not model-dependent. Moreover, various recent studies have also reported a correlation between changes in entanglement and digestion patterns from Limited Proteolysis Mass Spectrometry.^11,35^ Taken together, these results suggest that our coarse-grained model predictions are reliable.

## Conclusions

Protein folding in vivo is not solely regulated by the ribosome. Various other proteins and folding factors, such as chaperones, play a critical role in the process.^51−53^ In this study, we aimed to investigate the influence of the ribosome on protein folding alone. While it is commonly believed that the ribosome is generally effective in assisting protein folding to native conformations,^14,15,54,55^ our data do not consistently support this assumption. We do find the ribosome increases the folding efficiency of DHFR, in which two domains ABD and DLD fold independently. The ribosome confines the DLD domain inside the exit tunnel, allowing the ABD domain to fold cotranslationally and without interference; then, the DLD domain arranges into the correct native topology once released from the ribosome. In contrast, during refolding, all segments of the protein are simultaneously folding, presenting the opportunity for the formation of several non-native contacts between amino acids, thus enhancing the probability of being trapped in entangled misfolded states. For CAT-III and DDLB, which contain native entanglements, we did not observe an improvement in folding efficiency due to the ribosome, and in some cases, the ribosome caused these proteins to form intermediate misfolded states during cotranslational synthesis and these misfolded states persisted posttranslationally.

In conclusion, our findings suggest that the effect of ribosomes on protein folding is protein-specific and cannot be described by a universal rule. In general, the ribosome does not have a significant influence on folding outcomes.

## Supporting Information Available

Secondary structure diagrams of DHFR, CAT-III, and DDLB; fraction of native contacts versus time of ABD and DLD domains of DHFR; representative structures of the metastable states from clustering of DHFR; representative structures of the metastable states from clustering of CAT-III; representative structures of the metastable states from clustering of DDLB; mRNA templates used in continuous synthesis simulations; and structural definitions from pairs of secondary structures (PDF)
