# Supplementary material for: Is Posttranslational Folding More Efficient Than Refolding from a Denatured State: A Computational Study
Source: J Phys Chem B. 2023 May 18;127(21):4761–74. doi: 10.1021/acs.jpcb.3c01694 (PMC10240488; doi:10.1021/acs.jpcb.3c01694)
Supplement: Supplementary file 1 — jp3c01694_si_001.pdf [file jp3c01694_si_001.pdf]

Supplementary Information

Is Post-Translational Folding More Efficient Than Refolding from a Denatured State: A Computational Study

Quyen V. Vu[1], Daniel A. Nissley[2], Yang Jiang[3], Edward P. O'Brien[3, 4, 5*] and Mai Suan Li[1,6*]

[1]Institute of Physics, Polish Academy of Sciences; Al. Lotnikow 32/46, 02-668 Warsaw, Poland.

[2]Department of Statistics, University of Oxford, Oxford, OX1 3LB, UK.

[3]Department of Chemistry, Pennsylvania State University, University Park, PA 16802, USA.

[4]Bioinformatics and Genomics Graduate Program, The Huck Institutes of the Life Sciences, Pennsylvania State University, University Park, PA 16802, USA.

[5]Institute for Computational and Data Sciences, Pennsylvania State University, University Park, PA 16802, USA.

[6]Institute for Computational Sciences and Technology; Quang Trung Software City, Tan Chanh Hiep Ward, District 12, Ho Chi Minh City, Vietnam.

* To whom correspondence should be addressed: epo2@psu.edu, masli@ifpan.edu.pl

Supplementary Figures

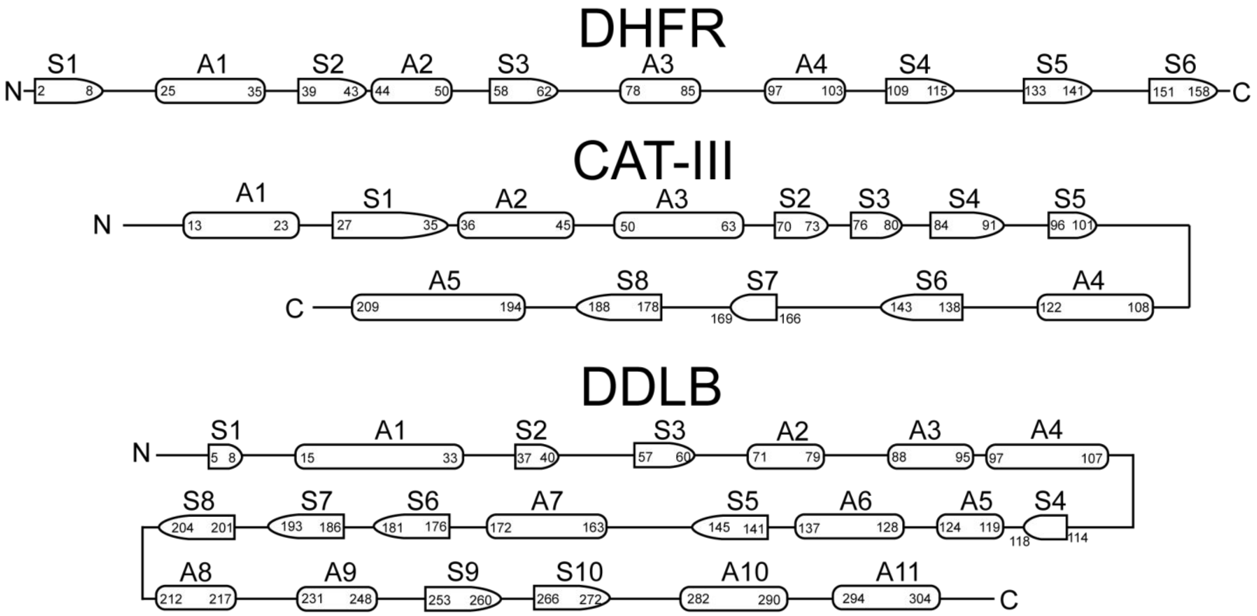

Figure S1: Secondary structure elements in DHFR, CAT-III, and DDLB. α-helices and β-sheets are labeled as A and S. Noted that the secondary structures are determined by Stride and we only show segments with more than 4 residues.

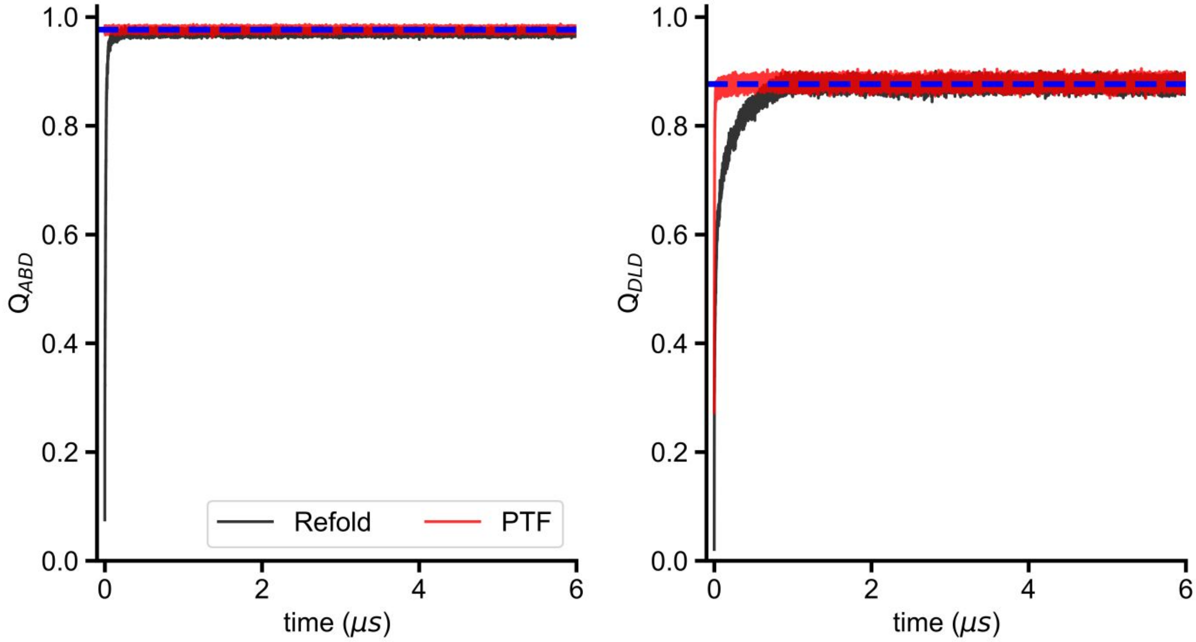

Figure S2: Fraction of native contacts versus time computed from folded trajectories of DHFR protein (un-normalized). (Left): ABD domain, (right) DLD domain. The blue-dashed line presents the threshold for each domain determined from the native simulation.

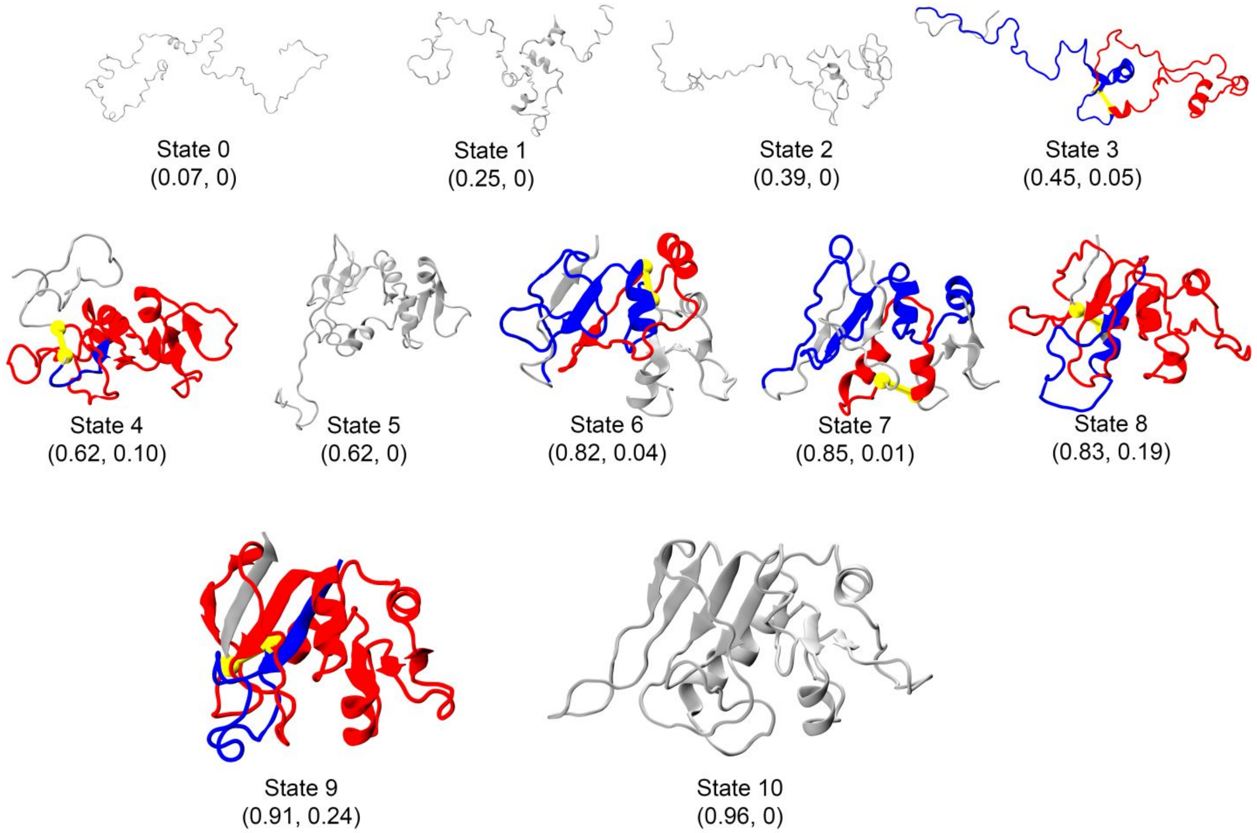

Figure S3: Representative structures of the metastable states from clustering of DHFR. The all-atom backmapped structures are presented with (Q, G) values. Entanglement in structure (if exists) is highlighted with the loop (red color) and is closed by the native contact (yellow sphere and yellow bond), and the threading segment is colored in blue. The metastable state indices and the (Q, G) values of the most probable microstates in that metastable state are shown below each structure.

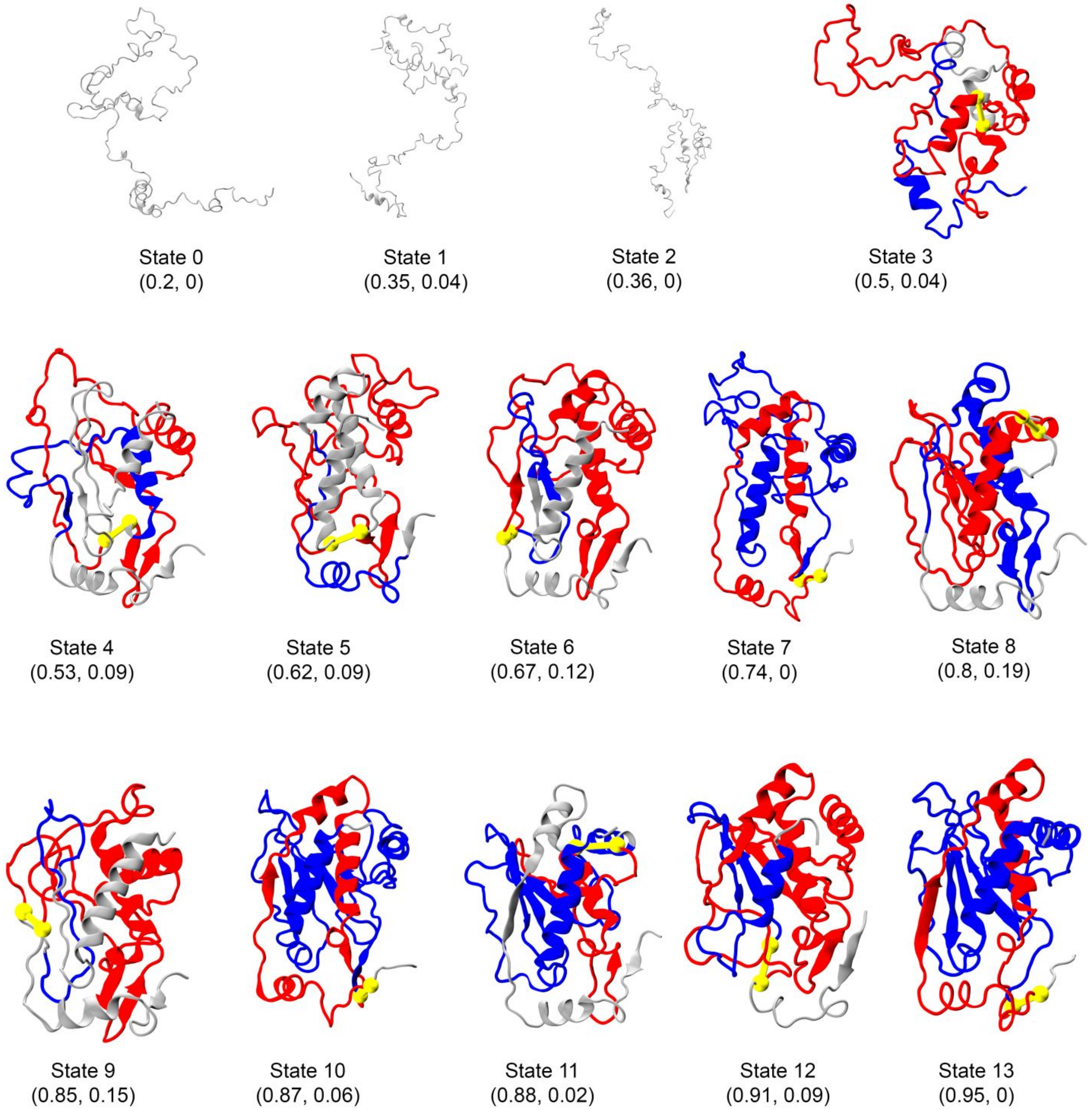

Figure S4: Representative structures of the metastable states from clustering of CAT-III. The all-atom backmapped structures are presented with (Q, G) values. Entanglement in structure (if exist) is highlighted with the loop (red color) and is closed by the native contact (yellow sphere and yellow bond), and the threading segment is colored in blue. The metastable state indices and the (Q, G) values of the most probable microstates in that metastable state are shown below each structure.

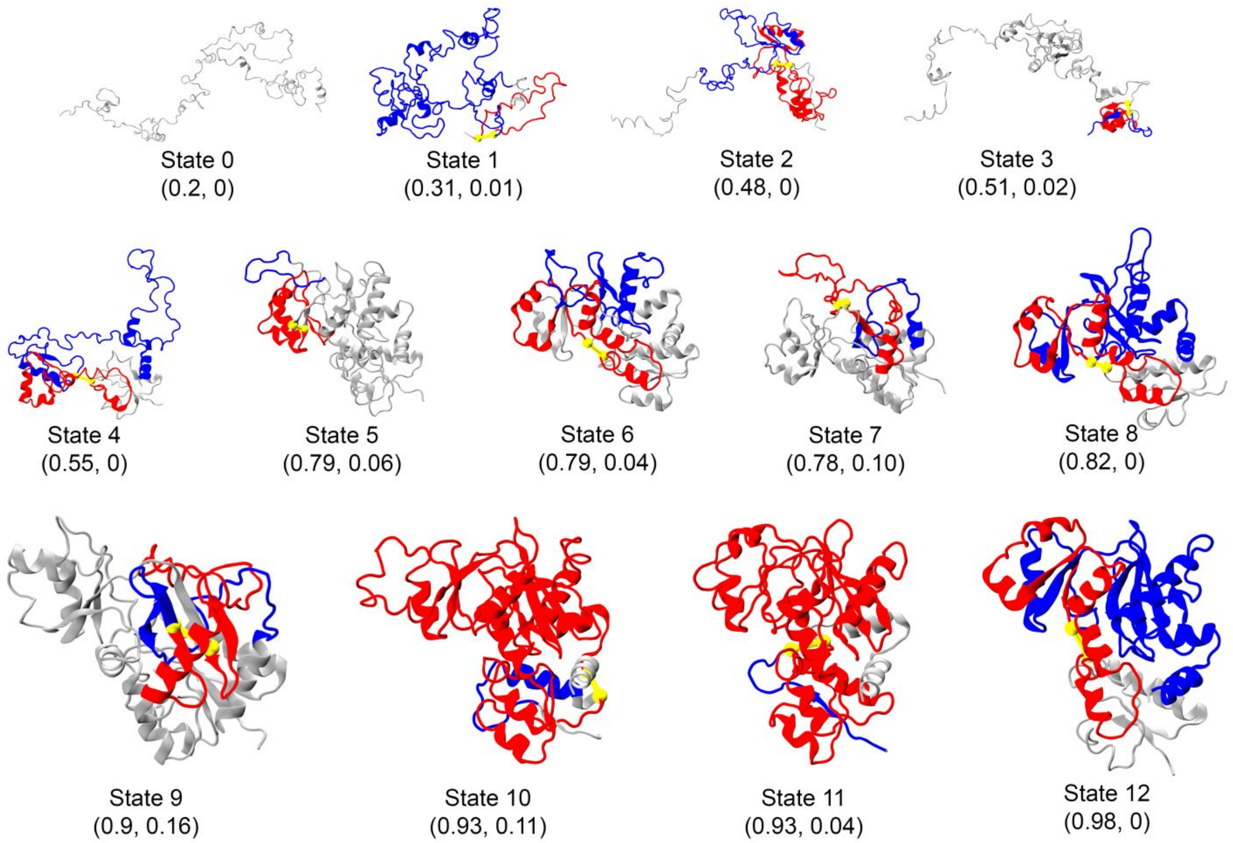

Figure S5: Representative structures of the metastable states from clustering of DDLB. The all-atom backmapped structures are presented with (Q, G) values. Entanglement in structure (if exist) is highlighted with the loop (red color) and is closed by the native contact (yellow sphere and yellow bond), and the threading segment is colored in blue. The metastable state indices and the (Q, G) values of the most probable microstates in that metastable state are shown below each structure.

Supplementary Tables

Table S1: Wild-type mRNA template for three enzymes in this study.

**Protein**	**mRNA template - Wildtype translation**
DHFR	AUGAUCAGUCUGAUUGCGGCGUUAGCGGUAGAUCGCGUUAUCGGCAUGGAAAACG<br>CCAUGCCGUGGAACCUGCCUGCCGAUCUCGCCUGGUUUAAACGCAACACCUUAAA<br>UAAACCCGUGAUUAUGGGCCGCCAUACCUGGGAAUCAAUCGGUCGUCCGUUGCCA<br>GGACGCAAAAAUAUUAUCCUCAGCAGUCAACCGGGUACGGACGAUCGCGUAACGU<br>GGGUGAAGUCGGUGGAUGAAGCCAUCGCGGCGUGUGGUGACGUACCAGAAAUCAU<br>GGUGAUUGGCGGCGGUCGCGUUUAUGAACAGUUCUUGCCAAAAGCGCAAAAACUG<br>UAUCUGACGCAUAUCGACGCAGAAGUGGAAGGCGACACCCAUUUCCCGGAUUACG<br>AGCCGGAUGACUGGGAAUCGGUAUUCAGCGAAUUCCACGAUGCUGAUGCGCAGAA<br>CUCUCACAGCUAUUGCUUUGAGAUUCUGGAGCGGCGGUAA

CAT-III	AUGAACUAUACAAAAUUUGAUGUAAAAAAUUGGGUUCGCCGUGAGCAUUUUGAGUU<br>UUAUCGGCAUCGUUUACCAUGUGGUUUUAGCUUAACAAGCAAAAUUGAUAUCACGA<br>CGUUAAAAAAGUCAUUGGAUGAUUCAGCGUAUAAGUUUUAUCCGGUAAUGAUCUAU<br>CUGAUUGCUCAGGCCGUGAAUCAAUUUGAUGAGUUGAGAAUGGCGAUAAAAGAUG<br>AUGAAUUGAUCGUAUGGGAUUCAGUCGACCCACAAUUCACCGUAUUCCAUCAAGAA<br>ACAGAGACAUUUUCAGCACUGAGUUGCCCAUACUCAUCCGAUAUUGAUCAAUUUAU<br>GGUGAAUUAUUUAUCGGUAAUGGAACGUUAUAAAAGUGAUACCAAGUUAUUUCCUC<br>AAGGGGUAACACCAGAAAAUCAUUUAAAUAUUUCAGCAUUACCUUGGGUUAAUUUU<br>GAUAGCUUUAAUUUAAAUGUUGCUAAUUUUACCGAUUAUUUUGCACCCAUUAUAAC<br>AAUGGCAAAAUAUCAGCAAGAAGGGGAUAGACUGUUAUUGCCGCUCUCAGUACAGG<br>UUCAUCAUGCAGUUUGUGAUGGCUUCCAUGUUGCACGCUUUAUUAAUCGGCUACA<br>AGAGUUGUGUAACAGUAAAUUAAAAUAA
DDLB	AUGACUGAUAAAAUCGCGGUCCUGUUGGGUGGGACCUCCGCUGAGCGGGAAGUUU<br>CUCUGAAUUCUGGCGCAGCGGUGUUAGCCGGACUGCGUGAAGGCGGUAUUGACG<br>CGUAUCCUGUCGACCCGAAAGAAGUCGACGUGACGCAACUGAAGUCGAUGGGCUU<br>UCAGAAAGUGUUUAUCGCGCUACACGGUCGCGGCGGUGAAGAUGGUACGCUGCAG<br>GGGAUGCUCGAGCUGAUGGGCUUGCCUUAUACCGGAAGCGGAGUGAUGGCAUCU<br>GCGCUUUCAAUGGAUAAACUACGCAGCAAACUUCUAUGGCAAGGUGCCGGUUUAC<br>CGGUCGCGCCGUGGGUAGCGUUAACCCGCGCAGAGUUUGAAAAAGGCCUGAGCGA<br>UAAGCAGUUAGCAGAAAUUUCUGCUCUGGGUUUGCCGGUUAUCGUUAAGCCGAGC<br>CGCGAAGGUUCCAGUGUGGGAAUGUCAAAAGUAGUAGCAGAAAAUGCUCUACAAGA<br>UGCAUUAAGAUUGGCAUUUCAGCACGAUGAAGAAGUAUUGAUUGAAAAAUGGCUAA<br>GUGGGCCGGAGUUCACGGUUGCGAUACUCGGUGAAGAAAUUUUACCGUCAAUACG<br>UAUUCAACCGUCCGGAACCUUCUAUGAUUAUGAGGCGAAGUAUCUCUCUGAUGAGA<br>CACAGUAUUUCUGCCCCGCAGGUCUGGAAGCGUCACAAGAGGCCAAUUUGCAGGC<br>AUUAGUGCUGAAAGCAUGGACGACGUUAGGUUGCAAAGGAUGGGGACGUAUUGAC<br>GUUAUGCUGGACAGCGAUGGACAGUUUUAUCUGCUGGAAGCCAAUACCUCACCGG<br>GUAUGACCAGCCACAGCCUGGUGCCGAUGGCGGCACGUCAGGCAGGUAUGAGCUU<br>CUCGCAGUUGGUAGUACGAAUUCUGGAACUGGCGGACUAA

Table S2: Structural definitions as the pair of secondary structures (shown in Figure S1) in the native state of proteins.

Protein	Pair		#native contacts	Pair		#native contacts	Pair		#native contacts
DHFR	**C1**	S_5-S_6	30	**C6**	S_1-A_4	3	**C11**	A_1-S_2	2
	**C2**	S_4-S_6	25	**C7**	S_2-A_2	3	**C12**	S_2-A_3	2
	**C3**	S_1-S_4	21	**C8**	S_2-A_4	3	**C13**	A_3-A_4	2
	**C4**	S_2-S_3	16	**C9**	A_2-S_3	3			
	**C5**	S_3-A_3	7	**C10**	S_1-A_1	2			
CAT-III	**C1**	S_1-S_8	32	**C6**	S_6-S_7	12	**C11**	A_3-S_4	4
	**C2**	S_4-S_5	22	**C7**	A_3-A_5	9	**C12**	A_3-A_4	4
	**C3**	S_4-S_6	16	**C8**	A_2-S_8	7	**C13**	A_3-S_6	2
	**C4**	S_7-S_8	14	**C9**	A_2-A_5	7	**C14**	S_5-A_4	2
	**C5**	S_2-S_3	13	**C10**	S_2-A_5	4			
DDLB	**C1**	S_7-S_9	26	**C11**	S_5-A_7	6	**C21**	A_4-S_{10}	3
	**C2**	S_9-S_{10}	24	**C12**	A_3-S_{10}	5	**C22**	S_4-A_5	3
	**C3**	S_4-S_6	16	**C13**	S_4-A_6	5	**C23**	A_6-S_6	3
	**C4**	S_5-S_6	16	**C14**	S_7-A_{10}	5	**C24**	A_5-S_6	2
	**C5**	A_1-A_{11}	15	**C15**	A_{10}-A_{11}	5	**C25**	S_7-A_9	2
	**C6**	S_7-S_8	13	**C16**	S_1-A_1	4	**C26**	S_8-A_{10}	2
	**C7**	S_1-S_2	12	**C17**	A_2-A_3	4	**C27**	A_9-S_9	2
	**C8**	S_1-S_3	12	**C18**	A_3-A_4	4	**C28**	A_9-S_{10}	2
	**C9**	A_1-S_2	7	**C19**	A_7-S_6	4			
	**C10**	A_4-A_9	6	**C20**	A_3-A_9	3			
